# *In vivo* assessment of the elastic properties of women’s pelvic floor during pregnancy using shear wave elastography: design and protocol of the ELASTOPELV study

**DOI:** 10.1186/s12891-020-03333-y

**Published:** 2020-05-15

**Authors:** Bertrand Gachon, Xavier Fritel, Fabrice Pierre, Antoine Nordez

**Affiliations:** 1 Department of obstetrics and gynecology, Poitiers university hospital, 2 rue de la Miletrie CS90577, 86021 Poitiers Cedex, France; 2 Nantes Université, Movement - Interactions – Performance, MIP, EA 4334, F-44000 Nantes, France; 3 Poitiers University, INSERM, Poitiers university hospital, CIC 1402, Poitiers, France; 4 INSERM, Center for Research in Epidemiology and Population Health (CESP), U1018, Gender, Sexuality and Health Team, University Paris-Sud, UMRS 1018, Orsay, France; 5 Health and Rehabilitation Research Institute, Faculty of Health and Environmental Sciences, Auckland University of Technology, Auckland, New Zealand

**Keywords:** Perineal trauma, Shear wave elastography, Pregnancy, Levator ani muscle, Anal sphincter, Childbirth, Obstetric anal sphincter injury

## Abstract

**Background:**

Animal studies have reported an increase in pelvic floor muscle stiffness during pregnancy, which might be a protective process against perineal trauma at delivery. Our main objective is to describe the changes in the elastic properties of the pelvic floor muscles (*levator ani,* external anal sphincter) during human pregnancy using shear wave elastography (SWE) technology. Secondary objectives are as follows: i) to look for specific changes of the pelvic floor muscles compared to peripheral muscles; ii) to determine whether an association between the elastic properties of the *levator ani* and perineal clinical and B-mode ultrasound measures exists; and iii) to provide explorative data about an association between pelvic floor muscle characteristics and the risk of perineal tears.

**Methods:**

Our prospective monocentric study will involve three visits (14–18, 24–28, and 34–38 weeks of pregnancy) and include nulliparous women older than 18 years, with a normal pregnancy and a body mass index (BMI) lower than 35 kg.m^− 2^. Each visit will consist of a clinical pelvic floor assessment (using the Pelvic Organ Prolapse Quantification system), an ultrasound perineal measure of the anteroposterior hiatal diameter and SWE assessment of the *levator ani* and the external anal sphincter muscles (at rest, during the Valsalva maneuver and during pelvic floor contraction), and SWE assessment of both the *biceps brachii* and the *gastrocnemius medialis* (at rest, extension and contraction). We will collect data about the mode of delivery and the occurrence of perineal tears. We will investigate changes in continuous variables collected using the Friedman test. We will look for an association between the elastic properties of the *levator ani* muscle and clinical / ultrasound measures using a Spearman test at each trimester. We will investigate the association between the elastic properties of the pelvic floor muscles and perineal tear occurrence using a multivariate analysis with logistic regression.

**Discussion:**

This study will provide original in vivo human data about the biomechanical changes of pregnant women’s pelvic floor. The results may lead to an individualized risk assessment of perineal trauma at childbirth.

**Trial registration:**

This study was registered on https://clinicaltrials.gov on July 26, 2018 (NCT03602196).

## Background

Perineal trauma is a frequent complication of childbirth, which may lead to several pelvic floor disorders, such as anal incontinence, urinary incontinence, pelvic organ prolapse and sexual dysfunction [1–4] In the most severe cases, perineal trauma could involve an obstetric anal sphincter injury (OASI) (rupture of the external anal sphincter and, worse still, opening of the rectal mucosae) and/or levator avulsion. OASI, which occurs in nearly 5% of first deliveries, is associated with postnatal anal incontinence and dyspareunia [1]. Levator avulsion, which occurs in nearly 10% of first deliveries, is associated with pelvic organ prolapse and sexual dysfunction [3]. These injuries are associated with trauma of the pelvic floor muscles at vaginal delivery during which these muscles are overstretched, up to three times their initial length [5]. Several risk factors are described in the literature (forceps delivery, fetal macrosomia, etc.) However, the occurrence of these complications remains very difficult to predict [1–4]. It is likely that the risk of pelvic floor trauma can be influenced by intrinsic characteristics of the pelvic floor muscles and their ability to lengthen sufficiently to enable passage of the fetus through the birth canal without being damaged. Identifying women with a high risk of perineal trauma antenatally would enable clinicians to propose individualized counseling and preventive strategies for these women.

Few studies have indicated that some intrinsic biomechanical characteristics of pregnant women could be associated with the risk of perineal trauma [6–8]. In a recent prospective study, we reported an association between peripheral ligamentous laxity (assessed at the metacarpophalangeal joint) and the risk of OASI. In that study, the women with the greatest ligamentous laxity had the greatest risk of OASI [8]. This result supports the hypothesis of an association between a woman’s individual biomechanical characteristics and her risk of perineal trauma. However, the main limitation of this study was that it was designed to analyze data about an upper limb joint, which is probably very different from pelvic floor muscle tissues [8].

Data about changes in intrinsic characteristics of women’s pelvic floor muscles during pregnancy has mainly originated from experiments on rats [9–11]. Some authors have reported that an increase in muscular fiber length and an increase in pelvic floor muscle stiffness occurs during pregnancy, while no changes were reported for peripheral muscles [9–11]. This could be explained by the increase in mechanical loading (force due to gravity of growing fetus) applied to pelvic floor muscles during pregnancy [10]. This increase in elastic modulus may be a protective process from perineal trauma. On rats, studies reported an increase during pregnancy in both fiber length and stiffness measured at a given sarcomere length [9, 10]. These changes could be interpreted consequently from the increase of loading. The increase in fiber length was thought as a mechanism to limit the fiber strain that can cause injury. The increase in stiffness was thought to be related to extracellular matrix content and would likely reduce the risk of injury due to large strain that occurs during parturition [5, 9]. This is supported by a higher ultimate stress in biological tissues that have higher stiffness [12]. These data about animal experimentation need to be read with caution because there is no data proven that these phenomena occur in a same way in women.

To date, several techniques have been described to assess the in vivo elastic properties of the pelvic floor muscles (vaginal elastometry, tactile imaging, elastography) [13, 14]. One of the most relevant techniques is shear wave elastography (SWE), which allows a direct, quantitative and noninvasive assessment of the muscles [15]. Recently, we reported the feasibility of an in vivo assessment of the elastic properties of the *levator ani* muscle using this technique (100% of procedures allowing a visualization of the levator ani muscle and a measure of elastic properties in women with a lower than 35Kg.m^− 2^ body mass index) [13].

In accordance with animal experimentation, we hypothesize that there are changes in elastic properties of women’s pelvic floor through pregnancy and that SWE is relevant to follow these changes [9, 10]. They might be specific to pelvic floor muscles without, or less, significant changes for peripheral muscles such as *biceps brachii* or *gastrocnemius medialis*. Finally, the hypothetical changes in elastic properties of women’s pelvic floor may be associated with their intrinsic risk of perineal trauma at childbirth. Indeed, during vaginal delivery, a major strain is applied to pelvic floor muscles which are stretched up to 3 times their initial length [5, 16]. Thus, intrinsic elastic properties of pelvic floor muscles may be associated with their ability to support this strain without being damaged.

Our main objective is to describe the changes in the elastic properties of the pelvic floor muscles (*levator ani,* external anal sphincter) during human pregnancy using shear wave elastography (SWE) technology. The secondary objectives are as follows: i) to look for specific changes of the pelvic floor muscles compared to peripheral muscles; ii) to determine whether an association between the elastic properties of the *levator ani* and perineal clinical and B-mode ultrasound measures exists; and iii) to provide explorative data about an association between pelvic floor muscle characteristics and the risk of perineal tears.

## Methods

### Design

**Fig. 1:**
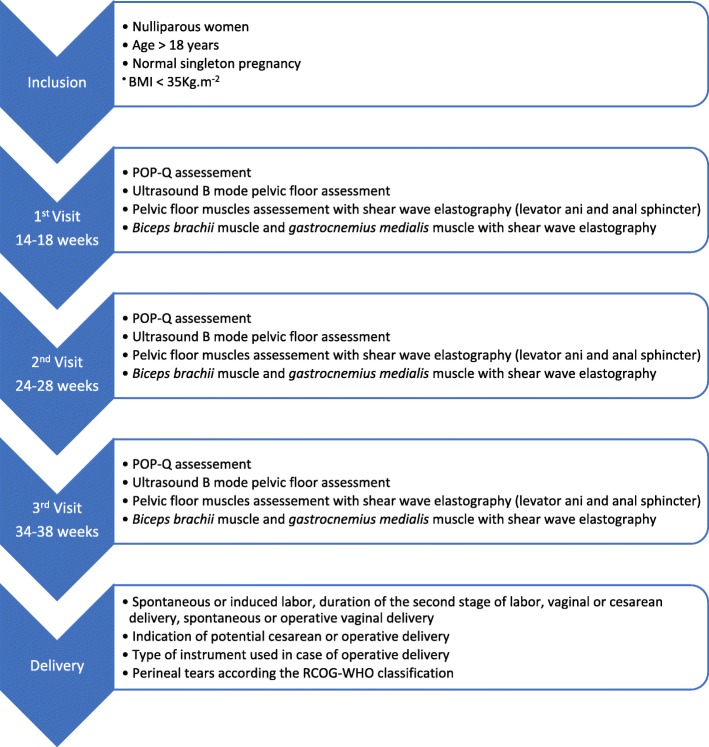
ELASTOPELV study design, inclusion criteria and data collected

The ELASTOPELV study will be a prospective, longitudinal, monocentric study. The scheme of the study will involve 3 visits during pregnancy: the first one between 14 and 18 weeks, the second between 24 and 28 weeks and the last between 34 and 38 weeks of pregnancy (Fig. 1). For each of these three visits, the protocol will follow these steps: clinical perineal assessment, ultrasound B-mode perineal assessment, SWE assessment of the *levator ani* muscle, the external anal sphincter, the *biceps brachii* muscle and the *gastrocnemius medialis* muscle (Fig. 1).

### Setting

The study will take place in the department of Obstetrics and Gynecology of the Poitiers University Hospital, Poitiers, France.

### Population

The inclusion criteria are as follows: women older than 18 years, volunteers, nulliparous, with a normal singleton pregnancy, and who benefit from health insurance.

The exclusion criteria are as follows: women with previous vaginal and/or cesarean delivery, women with a personal history of pelvic floor disorders (urinary incontinence, anal incontinence, pelvic organ prolapse), women with a body mass index (BMI) higher than 35 kg.m^− 2^, women with chronic muscular disease, women requiring admission into a psychiatric unit, women under judicial protection, and women unable to understand the French language.

If an included woman has a pregnancy who became pathological (define by the necessity of follow-up into pathological pregnancies consultations and/or admission in pathological pregnancies unit) she will no longer participate to the study and no data will be collected after this event. If a woman wants to stop its participation, no more data will be collected.

### Power calculation

This study deals with exploratory data with an absence of previous data that would allow a power calculation. Furthermore, the main endpoint of the project is descriptive (to describe changes in the elastic properties of the *levator ani* muscle during pregnancy), and, therefore, an a priori power calculation does not appear necessary. We aim to obtain and study the data from at least 50 women. We considered this sample size in part due to previous studies that reported an increase in *levator hiatus* area and ligamentous laxity during pregnancy, as well as changes in the intrinsic biomechanical characteristics of pregnant women, from between 20 to 50 women [17–19]. We estimate that 20% of the women will be excluded during pregnancy because of a complicated pregnancy and/or their own choice, leading to an objective of 60 inclusions.

### Recruiting procedure

Women eligible for the ELASTOPELV study will be informed about the study during their clinical consultations and/or ultrasound consultations during a normal pregnancy follow-up by their obstetrician and/or midwife. Eligible women interested in this study will be contacted by the investigator to obtain more information about the study and proceed with the inclusion if they give their free informed consent.

### Shear wave elastography principles

The novelty of the ELASTOPELV study is based on the use of SWE to investigate the in vivo elastic properties of the pelvic floor muscles of pregnant women. SWE allows a quantitative in vivo assessment of tissues during a classic ultrasound examination [15, 20]. An Aixplorer® device (Supersonic Imagine, Aix-en-Provence, France) will be used. A remote mechanical perturbation is applied to the tissue using a specific ultrasound sequence to induce the propagation of a shear wave into the tissue of interest. Due to the ultrafast ultrasound acquisition, the wave’s propagation speed is measured perpendicular to the ultrasound beam. This shear wave speed propagation is linked with the elastic modulus of the tissue: the stiffer the tissue, the higher the wave’s propagation speed is [15, 20, 21]. The elastic properties of the tissue are reported as the Young modulus, which represents the link between a stress and a strain in an isotropic tissue (similar mechanical properties in all directions). Muscles are stiffer along the fiber direction and thus cannot be considered isotropic. Considering an isotropic solid, the Aixplorer device gives E (Young’s modulus) as a measurement with, E = 3 μ = ρV^2^. with μ the shear modulus, ρ the density, V the shear wave speed.

In anisotropic solid the eq. E = 3 μ is no more valid. So, measurements should be divided by a factor 3 to obtain measurement of the muscle shear modulus [15, 22]. A previous study has demonstrated that the shear modulus is strongly and linearly related to the Young modulus, which supports the relevance of shear modulus measurements obtained with the Aixplorer® device for the study of muscle biomechanics [15, 23].

SWE is based on the hypothesis of a linear elasticity that is commonly assumed in both magnetic resonance elastography and ultrasound SWE. A lot of SWE studies analyzed the effects of loading on changes in muscle elasticity [15]. The effect of nonlinear elasticity should be studied in the future.

#### Safety

The protocol will be performed with a commercialized ultrasound scanner. This is considered a noninvasive and very safe examination [24]. The technology is widely used to assess the elastic properties of peripheral muscles without any adverse outcomes [15, 25]. Previous studies have reported the use of SWE during pregnancy for both mother and fetal tissue assessment without any adverse outcomes. Therefore, the use of SWE for the assessment of the pelvic floor muscles of pregnant women is safe [26, 27].

### Data collection

#### Women’s characteristics

At the first visit, after validation of the inclusion and exclusion criteria, we will collect anthropometric data about the women: height (in cm), weight (in kg) and BMI (in kg.m^− 2^). Demographic data and obstetric history will also be collected during the first visit: age (in years), gestity, and verification of the absence of a previous delivery (cesarean or vaginal). The dominant side will be recorded: right-handed or left-handed.

#### Clinical pelvic floor assessment

We will perform a clinical pelvic floor assessment at each visit. This examination will follow the recommendation of the Pelvic Organ Prolapse Quantification system (POP-Q) [28]. We will perform the procedure with women in the lithotomy position after voiding and maximal strain on the Valsalva maneuver. The position of each point of the POP-Q will be expressed in negative or positive values (in cm), and the length of each segment of the POP-Q (genital hiatus (gh), perineal body (pb), total vaginal length (tvl)) will also be expressed in centimeters [28].

#### Ultrasound B-mode pelvic floor assessment

We will perform an ultrasound B-mode pelvic floor assessment at each visit of the study. This examination is performed with the woman in the lithotomy position after voiding. We will use an Aixplorer® device with an XC6–1 1–6 MHz abdominal curved probe (V12, Supersonic Imagine, France). We will measure the anteroposterior hiatal diameter (distance between the antero-inferior extremity of the pubic symphysis and the anorectal junction, in cm) at rest, during a maximal strain on the Valsalva maneuver and at maximal perineal contraction. For these measures, we will use the translabial perineal ultrasound approach widely described by Dietz et al. [29, 30]. We will ask women to perform two initial Valsalva maneuvers with biofeedback instruction to prevent levator coactivation from serving as a confounding factor in our analysis [31].

#### Shear wave elastography assessments

As previously stated, an assessment of the elastic properties of the pelvic floor muscles of pregnant women will be performed at each visit using SWE. These measures will be performed for the *levator ani,* the external anal sphincter, *biceps brachii and gastrocnemius medialis* muscles. Each measurement will be performed on the right side of the woman, as it would be ideal to obtain all the measurements for the same side, preferentially while the women are in left lateral decubitus, which offers the possibility of accessing the right limbs. For each muscle’s location, we will investigate the muscle during three conditions: rest, stretch and subjective maximal contraction. We will use an Aixplorer® device (V12, Supersonic Imagine, France) with a linear SL 18–5 probe (5–18 MHz). Every measure will begin by performing a B-mode procedure to locate the muscle. Then, we will proceed with recording a 10-s video clip of the SWE measurements. The region of interest will be outlined by hand and the measure of the shear modulus will be obtained within this region in postprocessing. For assessment at rest and during a stretch, we will consider the mean shear modulus of the video clip, whereas for assessment during contraction, we will consider the maximal shear modulus.

We will perform three measures for each condition (rest, stretch and contraction) and consider the mean of the three measures for analysis. We choose to consider the mean of the 3 acquisitions for each condition to maximize the reliability of the measurement by considering all the measures (the most intense and the weakest contraction, the first measure after installation, etc.)

As previously mentioned, we will measure the Young modulus using the Aixplorer® device, which will be divided by a factor of 3 to obtain the shear modulus, which is more accurate for anisotropic tissues such as muscle [23].

One single investigator will perform all the measurements.

- Specificity for the *levator ani* muscle assessment

**Fig. 2:**
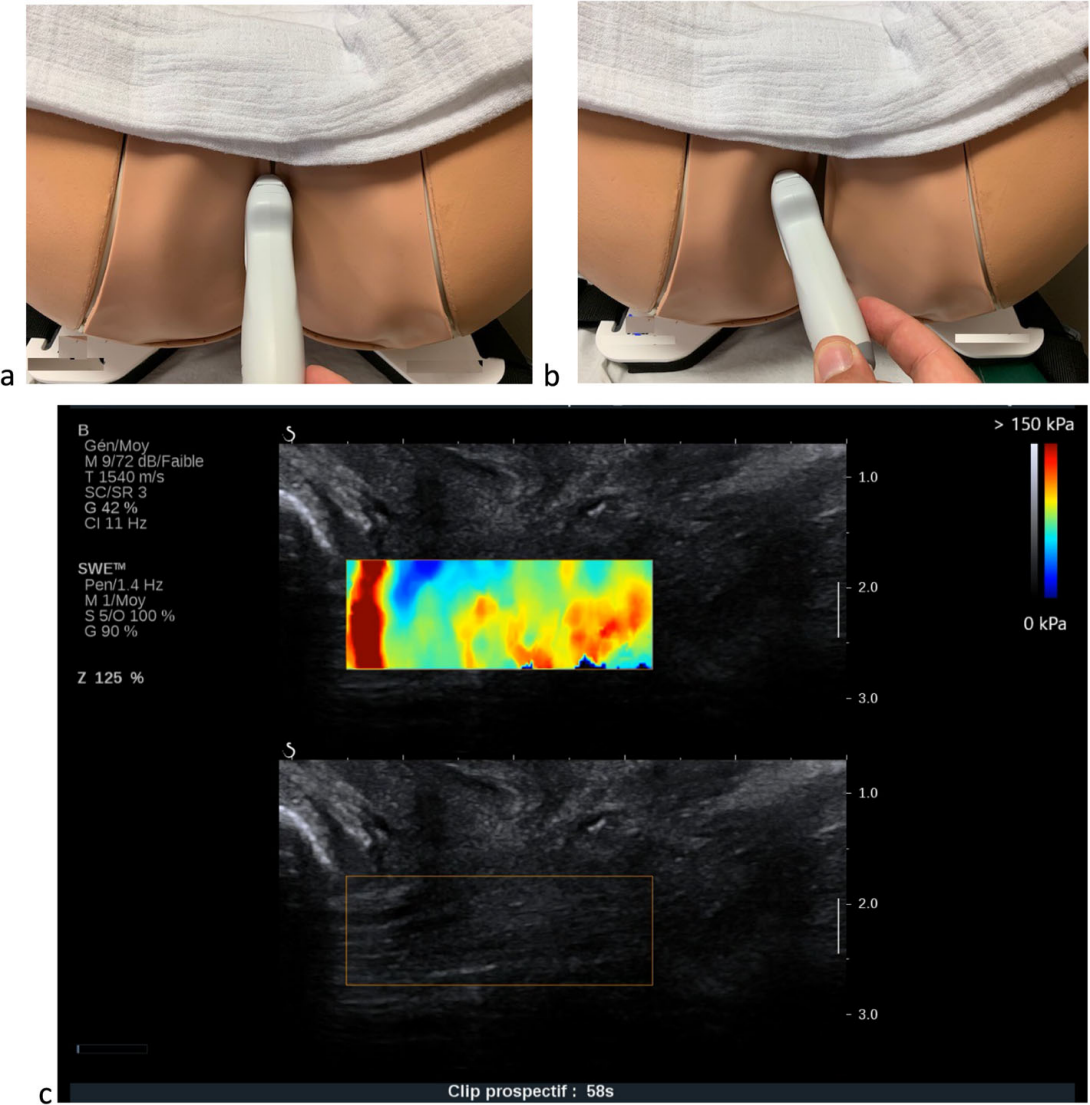
Levator ani muscle SWE assessment: probe position and example of acquisition

For this assessment, we will use the procedure that we described for nonpregnant women in a previous publication [13]. The examination will be performed with the woman in the lithotomy position after voiding. We will first locate the *levator ani* muscle at its pubic insertion during a B-mode ultrasound using the procedure described by Dietz et al. for the diagnosis of levator avulsion, with 87% agreement between observers. We will place the probe in the sagittal plane on the perineum and apply a 10° parasagittal inclination to identify the muscle (Fig. 2) [32]. We will perform assessments during the three considered conditions: rest, stretch and subjective maximal contraction. For the stretch condition, the woman will be asked to perform a maximal Valsalva maneuver. We will prevent levator coactivation in the same way that we described for ultrasound pelvic floor assessment [31]. With this procedure, a previous study reported that the shear modulus measured in *levator ani* muscle in non pregnant women is about 16 kPa at rest and 35 kPa during Valsalva maneuver [13].

- Specificity for external anal sphincter assessment

**Fig. 3:**
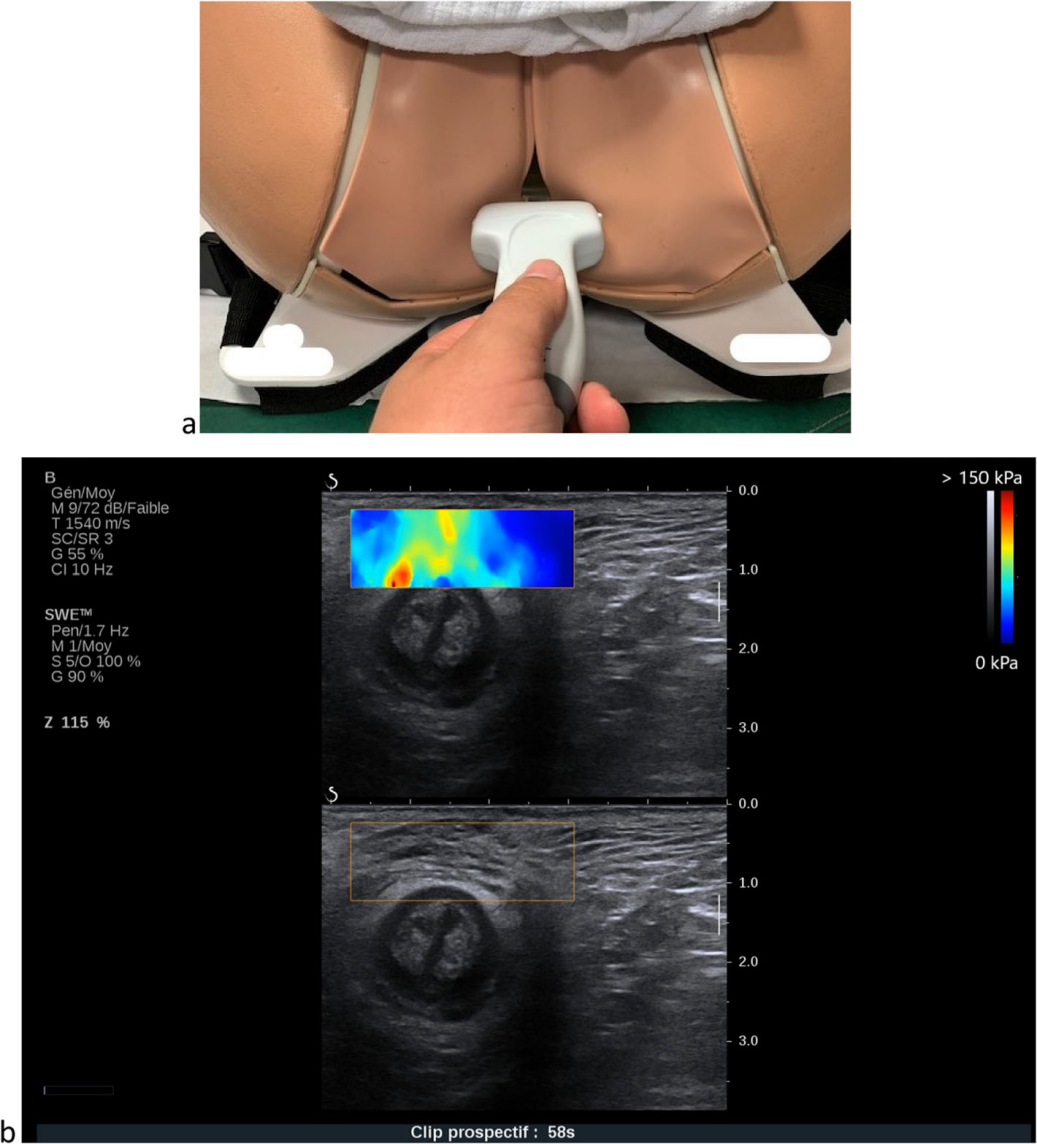
External anal sphincter SWE assessment: probe position and example of acquisition

The woman’s position will be the same as for the levator ani muscle. We will place the probe on the perineum immediately above the anus in the axial plane (Fig. 3). We will first locate the external muscle using a B-mode ultrasound and then proceed to the SWE assessments in the middle of the anterior zenith of the sphincter ring for the three conditions: rest, maximal Valsalva maneuver and subjective maximal perineal contraction [33].

- Specificity for biceps brachii muscle assessment

**Fig. 4:**
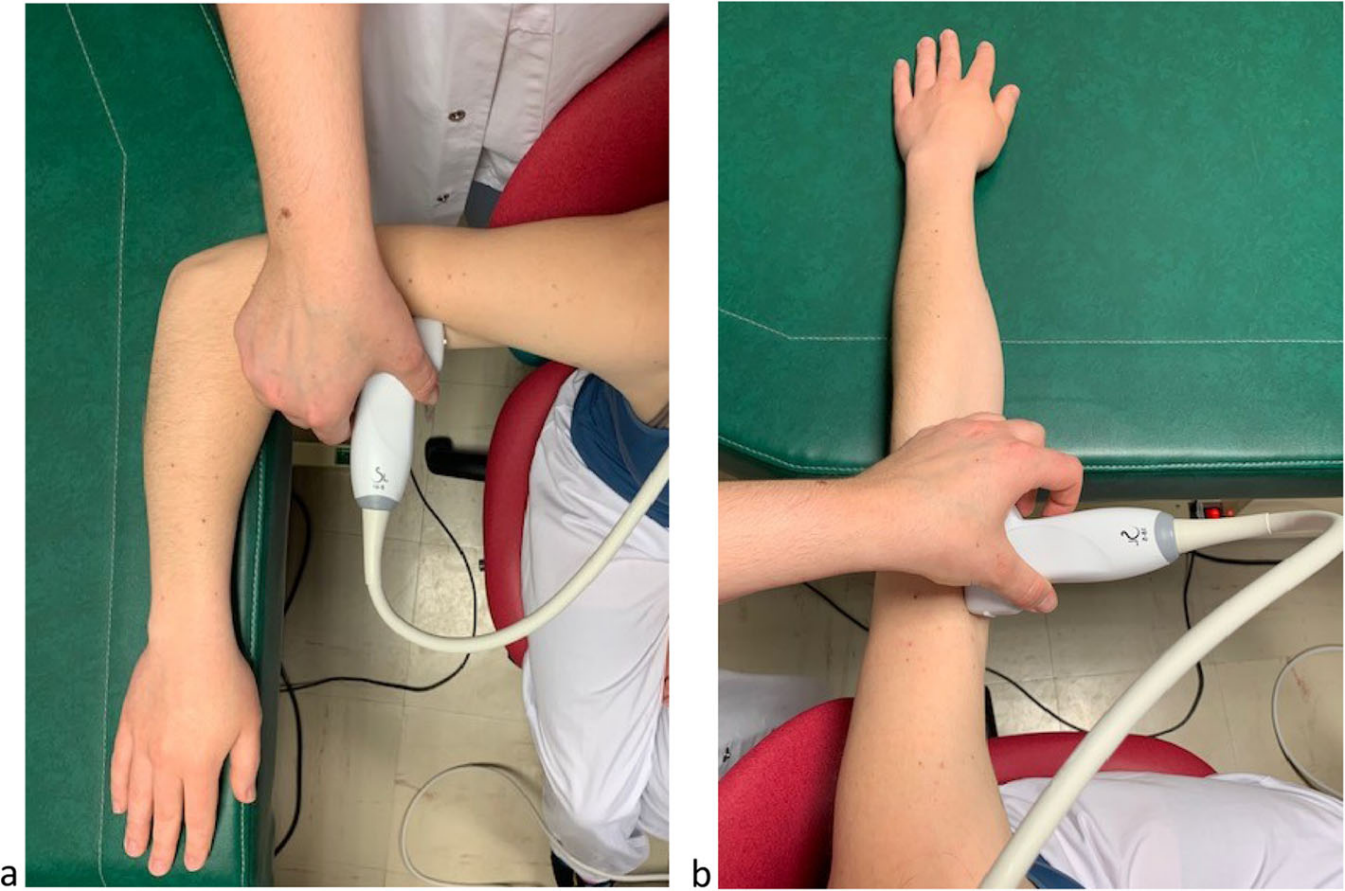
SWE acquisitions of the *biceps brachii* muscle at rest and standardized extension

First, we will identify the proximal and distal insertion of the biceps brachii using B-mode ultrasound and perform SWE acquisition midway between these insertions for three conditions: rest, standardized extension, and subjective maximal contraction. We will perform an assessment at rest performed with the upper limb having a 90° flexion of the elbow, which will be at the same height as the shoulder, with the hand in the pronation position. The forearm will rest on a flat support, allowing the *biceps brachii* to be totally free and accessible (Fig. 4A). We will systematically verify the 90° flexion of the elbow using a digital goniometer. For the assessment during extension, the position will be the same but with a 180° extension of the elbow (verified with the digital goniometer) and the hand in the pronation position (Fig. 4b). Finally, for the measurements during contraction, we will ask the woman to have a subjective maximal contraction of her *biceps brachii* in the rest assessment position. With this procedure, a previous study reported that the shear modulus measured in *biceps brachii* muscle in non pregnant volunteer is about 3 kPa at rest and 19 kPa when stretched [25, 34].

- Specificity for gastrocnemius medialis muscle assessment

**Fig. 5:**
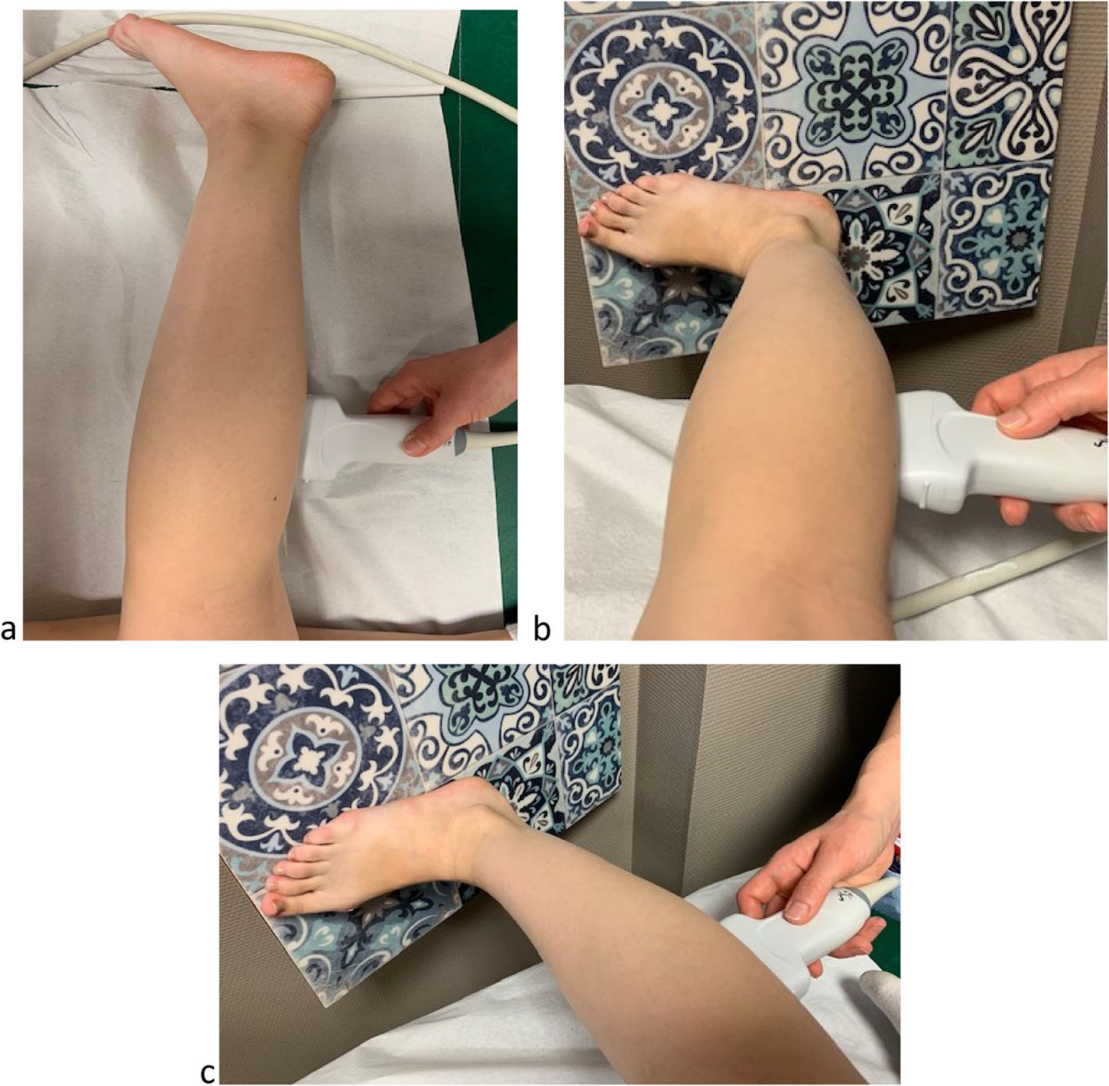
SWE acquisitions of the *gastrocnemius medialis* muscle at rest and standardized extension

Usually, this measure is performed with the volunteer lying down in ventral decubitus. Because of the evident risks of compression of the gravid uterus, such a position is not ideal for pregnant women, and so the assessments will be performed while the woman is in left lateral decubitus. First, we will identify the proximal and distal insertions as well as the lateral borders of the *gastrocnemius medialis* in B-mode ultrasound. We will perform the SWE acquisition midway between the lateral borders and midway between the proximal and distal insertions of the muscle for the three conditions: rest, standardized extension, and subjective maximal contraction. For the assessment at rest, the left leg will be flexed, the right leg will be fully extended (180°, verified with the digital goniometer) and the ankle will be in a neutral position (Fig. 5a). For the measurement during extension, the woman will be in the same position but with the right foot supported on a 20° inclined plane to apply a standardized extension of the *gastrocnemius medialis* (Fig. 5b). Finally, we will proceed to obtain the measurement during contraction with the woman in the same position as for the assessment at rest but with a voluntary maximal contraction of the *gastrocnemius medialis.* With this procedure, a previous study reported that the shear modulus measured in *gastrocnemius medialis* muscle in non pregnant volunteer is about 3.1 kPa at rest [25].

- *Data related to mode of delivery*

After childbirth, we will obtain the following data from the subjects’ medical files:

- spontaneous or induced labor
- epidural analgesia
- duration of the second stage of labor (time between full cervical dilatation and the birth, in minutes)
- duration of the expulsive phase (time between the onset of pushing and the birth, in minutes)
- mode of delivery (spontaneous vaginal delivery, operative vaginal delivery, cesarean delivery)
- indication for potential cesarean delivery (fetal distress, other)
- type of instrument used for potential operative delivery (vacuum, forceps, spatulas)
- indication for potential operative delivery (fetal distress, other)
- episiotomy use
- potential perineal tears classified according to the Royal College of Obstetricians and Gynaecologists (RCOG) guidelines [35, 36].

### Analysis

#### Judgment criteria

The primary judgment criteria will be the evolution of the shear modulus of the pelvic floor muscle (*levator ani* and external anal sphincter) across the pregnancy assessed at rest, during the Valsalva, and during a contraction.

Secondary judgment criteria will be:

- the association between POP-Q measurements (especially gh and pb) and the elastic properties (shear modulus) of the levator ani muscle at each visit;
- the association between perineal B-mode ultrasound measurements and the elastic properties (shear modulus) of the levator ani muscle at each visit;
- the changes in shear modulus of the *biceps brachii* and *gastrocnemius medialis* muscles during pregnancy compared to the pelvic floor muscles;
- the association among the shear modulus of the pelvic floor muscles (*levator ani* and external anal sphincter) at the last visit, the mode of delivery (spontaneous vaginal delivery, operative vaginal delivery, cesarean delivery) and the potential occurrence of a perineal tear (RCOG-WHO classification, French guidelines) [35, 36].

#### Plan of analysis

We will describe the anthropometric and sociodemographic characteristics of the included women. Age, BMI and term of pregnancy will be reported as the mean and standard deviation (SD). For all the other analysis, we will only consider data about women who completed the three planned visits. For each trimester, we will only collect continuous variables, which will be reported as the means and SDs. Changes in these variables during pregnancy will be investigated using a Friedman test. Obstetric data will be reported as the means and SD for continuous variables and as percentages and frequencies for categorical variables.

Regarding the study endpoints, we will first report the main outcome of this study, which consists of the changes in the pelvic floor muscle’s shear modulus across the pregnancy. Then, we will report the changes in all other measured pelvic floor-related parameters (POP-Q measurements, ultrasound B-mode measurements). We will look for an association between the shear modulus of the levator ani muscle and clinical (POP-Q measurements) and ultrasound B-mode assessments at each trimester using a Spearman correlation coefficient calculation. Second, we will report the changes in the shear modulus of the *biceps brachii* and *gastrocnemius medialis* muscles to look for changes between the different investigated locations. Third, we will look for an association between the shear modulus of the pelvic floor muscles (*levator ani* and external anal sphincter) and both the mode of delivery (vaginal or cesarean delivery) and perineal tear occurrence using univariate analysis. Variables with a level of significance greater than *p* < 0.15 in univariate analysis will be included in the multivariate analysis using a logistic regression. We will perform statistical analysis with Stata software (version V14IC; Stata Corporation, College Station, TX, USA). For all analyses, significance will be considered for *p* < 0.05, and we will calculate odds ratios (ORs) with 95% confidence intervals when appropriate.

### Study duration

We have planned for an 18-month period of inclusion, which led to a total study duration (from the inclusion of the first women to the end of the follow-up of the last women) of 24 months.

### Ethical and reglementary considerations

Every volunteer will receive oral and written information about the study and must give her free and informed written consent before any investigation. The study was approved by an ethical committee (*Comité de Protection des Personnes Ile de France VIII)* and is referenced with the ID RCB: 2018-A01422–53. The study is also registered on https://clinicaltrials.gov (NCT03602196).

### Availability of data and materials

Supporting data could be accessed on request to Poitiers University Hospital, Department of gynecology and Obstetrics, France.

## Discussion

### Short summary of the study

It is difficult to predict the outcome of severe perineal trauma (OASI and/or levator avulsion) at childbirth, as there is a strong potential of an alteration of the woman’s health. One hypothesis to optimize the efficiency of risk prediction is to consider the intrinsic biomechanical characteristics of women’s pelvic floors. Such an approach may allow an individualized risk assessment personalized information for each woman. Our prospective, monocentric, longitudinal study will include 60 nulliparous pregnant women. Three visits are planned in this protocol (one per trimester of pregnancy) and will include clinical (POP-Q) and ultrasound assessment of the pelvic floor, SWE assessment of the pelvic floor muscles (*levator ani,* external anal sphincter) and the *biceps brachii* and *gastrocnemius medialis* muscles. Finally, data about the mode of delivery (cesarean section or vaginal delivery) and the occurrence of perineal tears will be collected. The main endpoint will be to describe the changes in the elastic properties of the pelvic floor muscles across pregnancy. The secondary endpoints will be to look for an association between SWE measurements of the *levator ani* muscle and clinical and ultrasound perineal assessments, to compare muscular changes during pregnancy among the pelvic floor muscles and the *biceps brachii and gastrocnemius medialis* muscles and to look for an association among the elastic properties of the pelvic floor muscles, the mode of delivery and the occurrence of perineal tears.

### Justification of methodological choices

#### Choice of shear wave elastography technology to investigate pelvic floor muscles

Few other techniques have been proposed for investigating the elastic properties of pelvic floor muscles. Kruger et al. reported the use of a vaginal elastometer to investigate the elastic properties of the *levator ani* muscle in both pregnant and nonpregnant women [14, 37]. This device consists of a vaginal speculum with several force sensors, allowing the acquisition of a force/displacement curve. Such a device is quite interesting, but because it measures the properties of both the vaginal wall and the *levator ani* muscle, the measurements of the elastic properties of the *levator ani muscle* might be biased. Furthermore, we think that the vaginal intrusion could be associated with a lower participation rate since it involves the intromission of a medical device by an investigator. Egorov et al. developed a vaginal tactile imaging device consisting of a vaginal ultrasound probe supplemented with force and temperature sensors [38, 39] . Such a device is expected to provide an assessment of the pelvic floor elastic properties. We consider that this technique presents the same limitations as the vaginal elastometer of Kruger et al. [14, 37]. Static elastography is another ultrasound-related functional imaging technology that can be used to assess women’s pelvic floors with a non-invasive approach [40–43]. However, this technique has major limitations in providing non direct and qualitative assessments of the pelvic floor.

The choice of the transperineal approach to assess pelvic floor muscles is supported by an important literature reporting that such an approach is efficient (in terms of acceptability and reliability) to investigate pelvic floor muscles [29, 30, 32, 33]. In 2018, our research team published a feasibility study on the use of SWE to investigate the elastic properties of the *levator ani muscle* in nonpregnant women with this transperineal approach [13]. In this paper, we report that we were able to individualize the *levator ani* muscle and to measure a shear modulus in 100% of women with a lower than 35Kg.m^− 2^ BMI which allows to report the feasibility of the procedure. We consider that the fact that we investigate only the right *levator ani* muscle do not induce any bias considering that in this previous study, we reported that there are no differences between the elastic properties of the right and left *levator ani* muscles, assessed using SWE [13]. There is no published technique for investigating in vivo the elastic properties of the external anal sphincter. Considering the easy access to the external anal sphincter using ultrasound with a transperineal approach and the efficiency of SWE in other muscle applications, we consider that this choice is relevant [33]. In the future, this examination could be easily performed in the ultrasound follow-up of pregnant women. We do not have data about the reproducibility of pelvic floor muscles assessment using SWE. Nevertheless, considering the easy access to these muscles with a transperineal approach and the fact that SWE is a reliable tool for assessing peripheral muscles we expected a good reproducibility of the technique.

#### Choice of investigating biceps brachii and gastrocnemius medialis muscles

We expect to study muscles with different characteristics. Considering that the *biceps brachii* is not exposed to any increases in mechanical loading related to pregnancy, we expect to find a different pattern compared to pelvic floor muscles. The difference might be less pronounced for the *gastrocnemius medialis* since this muscle is exposed to an increase of loading due to the increase in weight that occurs during pregnancy. We also chose these two peripheral muscles because they are superficial, large and easily accessible muscles. Furthermore, we have data reporting that SWE is reliable to investigate these muscles with high performance reliability indicators [25].

#### Justification of inclusion and exclusion criteria

We choose to include only nulliparous women in this study. This choice is easily understandable by the willingness to avoid bias related to any previous obstetrical perineal trauma. The elastic properties of the pelvic floor muscles that we will report in this study will be solely related to the intrinsic characteristics of the woman and the changes induced by the pregnancy. We will also exclude women with a BMI above 35 kg.m^− 2^. This is due to the results of a feasibility study that reported difficulties in SWE assessments of the *levator ani* muscle during the Valsalva maneuver for women with high BMIs [13]. These difficulties were due to a loss of visibility of the *levator ani* during the maneuver using a superficial linear probe; the muscle became too deep to be clearly located.

### Expected results

#### Concordance with animal experimentations

As we mentioned in the background section, human data about the evolution of pelvic floor muscles during pregnancy are lacking. It has been reported in animal experiments that during pregnancy, the stiffness of the pelvic floor muscle increases due to a drastic increase in total collagen content [9–11]. As we mentioned it in the background section, these animal experimentation related data must be interpreted carefully considering that there is no work with a confirmation that these phenomena occur in a same way in pregnant women.

We expect to report a similar increase in the stiffness of the pelvic floor muscles during pregnancy in our study, which will support the data from animal experiments. Such an increase in stiffness might be a protective process from perineal trauma given that tissue with the lowest stiffness easily raises their plasticity threshold to a level beyond which irreversible damage occurs in the tissue [12].

One potential confounding factor for the interpretation is that we do not have data about the use of perineal stretching device such as Epi-No® during pregnancy. There is no data about the impact of such a practice on pelvic floor muscle elastic properties. Some works reports an increase in perineal extensibility, but it reports maximal vaginal compliance to the Epi-No® without data about a direct assessment of pelvic floor muscles stiffness [44, 45]. Furthermore, its use is not recommended in French guidelines [36]. This considered, we think that the risk of bias in our cohort is weak.

#### Concordance between SWE measurements and clinical / ultrasound measurements

We expect to report an association between the elastic properties of the *levator ani* muscle and the clinical and ultrasound assessments of pelvic floor distension. For the clinical assessment, we will investigate all POP-Q measurements but with a special interest in the gh and pb measurements that are performed during the Valsalva maneuver and that reflect pelvic floor distension that occurs during the maneuver. A similar association will be investigated for the ultrasound assessment (distance between the pubic symphysis and the anorectal angle). If we can report such a correlation between the elastic properties of the *levator ani* investigated using SWE and clinical / ultrasound pelvic floor distension, it will support the efficiency and the applicability of SWE for functional pelvic floor muscle assessments. We choose to focus on the *levator ani* muscle for this analysis given the well-reported association between levator hiatus and pelvic organ mobility [46].

#### Preliminary data about the hypothetical association between elastic properties of women’s pelvic floors and obstetric perineal trauma

Finally, we will look for a potential association between the elastic properties of the pelvic floor muscles and the occurrence of perineal tears as well as the mode of delivery. Due to the expected number of women, it will not be possible to conclude about such an association. The objective is to provide preliminary data about the distribution of pelvic floor muscles elastic properties according the stage of perineal tear. We expect that these preliminary data would allow the future implementation of a larger multicentric prospective study investigating the interest of including the elastic properties of the pelvic floor muscles in our risk prediction of perineal trauma. This is required to offer each pregnant woman personalized information and an individualized preventive strategy. One prospect might be a selective use of episiotomy in high-risk women considering that this intervention, in a biomechanical study, reduce the stress on the muscles and the force required to delivery successfully [47]. Our data might be helpful in providing a justification for implementing this type of study and offering the possibility of performing a power calculation.

## Data Availability

Not applicable. This is a study protocol.

## Abbreviations

BMI: Body Mass Index
Gh: Genital hiatus
OASI: Obstetric Anal Sphincter Injury
Pb: Perineal body
POP-Q: Pelvic Organ Prolapse Quantification
RCOG: Royal College of Obstetricians and Gynaecologists
SD: Standard Deviation
SWE: Shear Wave Elastography
Tvl: Total vaginal length
WHO: World Health Organization
